# Fuzhenghefuzhiyang Formula (FZHFZY) Improves Epidermal Differentiation *via* Suppression of the Akt/mTORC1/S6K1 Signalling Pathway in Psoriatic Models

**DOI:** 10.3389/fphar.2021.650816

**Published:** 2021-08-11

**Authors:** Yue Lu, Haiming Chen, Junhong Zhang, Bin Tang, Hongyu Zhang, Changju Ma, Xiaojuan Tang, Li Li, Jingjing Wu, Jianan Wei, Shaoping Li, Lei Yang, Ling Han, Chuanjian Lu

**Affiliations:** ^1^ State Key Laboratory of Dampness Syndrome of Chinese Medicine, The Second Affiliated Hospital of Guangzhou University of Chinese Medicine (Guangdong Provincial Hospital of Chinese Medicine), Guangzhou, China; ^2^ Guangdong Provincial Key Laboratory of Clinical Research on Traditional Chinese Medicine Syndrome, Guangzhou, China; ^3^ Guangdong Provincial Clinical Medicine Research Center for Chinese Medicine Dermatology, Guangzhou, China; ^4^ Guangdong-Hong Kong-Macau Joint Lab on Chinese Medicine and Immune Disease Research, Guangzhou University of Chinese Medicine, Guangzhou, China; ^5^ State Key Laboratory of Quality Research in Chinese Medicine, Institute of Chinese Medical Sciences, University of Macau, Taipa, China; ^6^ Guangzhou Youcare Biopharmaceutics Co., Ltd, Guangzhou, China

**Keywords:** psoriasis, Fuzhenghefuzhiyang formula, epidermal differentiation, Akt/mTORC1/S6K1 pathway, imiquimod

## Abstract

Psoriasis is a chronic proliferative skin disorder characterised by abnormal epidermal differentiation. The Fuzhenghefuzhiyang (FZHFZY) formula created by Chuanjian Lu, a master of Chinese medicine in dermatology, has been external used in the Guangdong Provincial Hospital of Chinese Medicine for the treatment of psoriasis, but its mechanisms of action against psoriasis remain poorly understood. This study involved an exploration of the effects of FZHFZY on epidermal differentiation and its underlying mechanisms in interleukin (IL)-17A/IL-22/interferon (IFN)-γ/tumour necrosis factor (TNF)-α–stimulated HaCaT cells and in a mouse model of imiquimod (IMQ)-induced psoriasis. Cell viability was assessed by MTT assay. Epidermal differentiation was detected by reverse-transcription polymerase chain reaction and western blotting. Histological evaluation of the skin tissue was performed *via* haematoxylin and eosin staining, and the Akt/mTORC1/S6K1 pathway was analysed by western blotting. FZHFZY inhibited proliferation and improved epidermal differentiation in IL-17A/IL-22/IFN-γ/TNF-α–induced HaCaT cells. FZHFZY ameliorated symptoms of psoriasis, regulated epidermal differentiation and inhibited phosphorylation of the Akt/mTORC1/S6K1 pathway in the skin of mice with imiquimod-induced psoriasis. Our results suggest that FZHFZY may exhibit therapeutic action against psoriasis by regulating epidermal differentiation *via* inhibition of the Akt/mTORC1/S6K1 pathway.

## Introduction

Psoriasis is a chronic inflammatory skin disease that affects nearly 3% of the world’s population, which equates to more than 125 million cases globally (Rendon and Schäkel, 2019; Korman, 2020). There is no western medical cure for psoriasis, and its recurrence after drug cessation limits the clinical efficacy of available non-curative treatments. The current treatments for psoriasis include glucocorticoids, vitamin D analogues, tretinoin drugs and calcineurin inhibitors, which can alleviate symptoms and achieve therapeutic effects. However, their long-term use leads to adverse effects, such as relapse and rebound, and the development of skin atrophy and hypercalcaemia, especially after irregular use or abuse of glucocorticoids (Heath et al., 2019; Islam et al., 2019; Mourad et al., 2019; van der Kraaij et al., 2019). Chinese medicine is widely accepted by patients with psoriasis in China because of its reliable curative effects and long history. Our systematic reviews have demonstrated that Chinese medicine therapies are effective for the treatment of psoriasis (Yu et al., 2007; Yu et al., 2013; Zhang et al., 2014; Zhang et al., 2016; Yu et al., 2017).

The excessive proliferation of keratinocytes in psoriatic skin lesions promotes the differentiation of granular and keratinised layers, and alters the expression of keratin and related genes, resulting in abnormal epidermal differentiation, such as hypokeratosis, a thick spinous layer, a reduced granular layer and a prominent epidermis (Jain et al., 2016; Szczerkowska-Dobosz et al., 2020). The mammalian target of rapamycin (mTOR) is a serine/threonine kinase that comprises two protein complexes, the mammalian target of rapamycin complex 1 and 2 (mTORC1 and mTORC2); notably, mTORC2 is the main downstream regulator of protein kinase B (Akt) (Laplante and Sabatini, 2012; Yu and Cui, 2016; Switon et al., 2017). Studies have reported overactivity of mTORC1 and its downstream molecule ribosomal protein S6 kinase 1 (S6K1) in the skin lesions of patients with psoriasis (Buerger et al., 2017; Rabanal-Ruiz and Korolchuk, 2018), and the highly active Akt/mTORC1/S6K1 pathway can lead to the abnormal differentiation of keratinocytes, which is generally recognized as an important pathological feature of psoriasis (Akinduro et al., 2016; Balato et al., 2017; Cai et al., 2019).

The human immortalised keratinocyte HaCaT is a commonly used cell that can express differentiation markers such as keratin, loricrin, involucrin and filaggrin (Johnston et al., 2013). When keratinocytes are stimulated by TNF-α, IFN-γ, IL-22 and IL-17A, they can express the inflammatory marker proteins related to psoriasis, such as chemokines and proinflammatory cytokines (Jiang et al., 2017; Jiang et al., 2019). The mouse model of psoriasis induced by IMQ is quite similar to human psoriasis vulgaris in its clinical manifestations, histopathology and immunological changes (Flutter and Nestle, 2013). Therefore, this study involved the use of IL-17A/IL-22/IFN-γ/TNF-α–induced HaCaT cells and mice with IMQ-induced psoriasis.

Fuzhenghefuzhiyang decoction (FZHFZY) was developed by Chuanjian Lu, a master of Chinese medicine in dermatology. According to the theory of traditional Chinese medicine, FZHFZY includes seven herbs: *Radix seu Herba Cynanchi Paniculati. Densefruit Pittany Root-bark, Fructus Cnidii, Rhizoma Smilacis Glabrae, Radix Rehmanniae Preparata., Radix Angelicae Sinensis* and *Pericarpium Punicae Granati* (Chen et al., 2020). This formula has been used as a topical application to treat patients with psoriasis at the Guangdong Provincial Hospital of Chinese Medicine. Our retrospective analysis from 2010 to 2017 found that FZHFZY treatment showed satisfactory outcomes in the improvement of clinical symptoms and in alleviation of itching in patients with psoriasis. However, the mechanism of action of FZHFZY in the treatment of psoriasis remains unknown.

This study was performed to observe the effects of FZHFZY on epidermal differentiation in IL-17A/IL-22/IFN-γ/TNF-α stimulated HaCaT cells and a mouse model of IMQ-induced psoriasis. We further explored the mechanism of action of FZHFZY in the treatment of psoriasis to provide a foundation for the development of FZHFZY into a safe and effective modern formula.

## Materials and Methods

### Reagents

Dulbecco’s modified Eagle’s medium (DMEM) and fetal bovine serum (FBS) were purchased from Gibco Laboratories (Grand Island, NY, United States; catalogue nos. 0030034DJ and 12483020). Dexamethasone (DEX) cream was obtained from Shenzhen Huarunsanjiu Pharmaceutical Factory (Shenzhen, Guangdong, China; catalogue no. H44024170). Imiquimod (IMQ) cream was obtained from Sichuan Mingxin Pharmaceutical Co. Ltd (Sichuan, China; catalogue no. H20030128). Recombinant Human interleukin (IL)-17A, IL-22, interferon (IFN)-γ and tumour necrosis factor (TNF)-α were purchased from Peprotech (Rocky Hill, NJ, United States; catalogue nos. 96-200-17-5, 96-200-22-2, 96-300-02-20 and 96-300-01A-10). 3-(4, 5-Dimethylthiazol-2-yl)-2, 5-diphenyltetrazolium bromide (MTT) was obtained from Sigma-Aldrich (St. Louis, MO, United States; catalogue no. M2128). Antibodies specific for p-Akt (pT308), Akt, p-mTOR (pS2448), mTOR, p-S6K1 (pS424), S6K1, keratin 5, keratin 14, keratin 15 and glyceraldehyde 3-phosphate dehydrogenase (GAPDH) were all purchased from Abcam (Cambridge, MA, United States; catalogue nos. ab38449, ab8805, ab109268, ab32028, ab131436, ab32529, ab8245, ab52635, ab119695 and ab52816). Anti-loricrin antibody was obtained from Thermo Fisher Scientific (Waltham, MA, United States; catalogue no. PA5-30583). Antibodies specific for filaggrin and involucrin were obtained from Cohesionbio Biosciences Ltd (Burlington, NC, United States; catalogue no. CPA4410 and CPA1624). Antibodies specific for p-S6 (pS240/244) and S6 were obtained from Cell Signaling Technology (Danvers, MA, United States; catalogue no. 5364S and 2217S).

### Plant Materials and Preparation of FZHFZY

The FZHFZY formula in this study comprised seven Chinese herbal components (Table 1). All plant materials were pharmacopoeia-grade and were obtained from Kangmei Pharmaceutical Company Ltd. (Guangzhou, Guangdong, China). The herbs were extracted with distilled water, and the extracts were concentrated to 5 g/ml and stored at 4°C for the study.

**TABLE 1:** Constituents of FZHFZY.

Linnean classification	Botanical origin	Ratio
*Radix seu Herba Cynanchi Paniculati*	*Cynanchum panniculatum* (Bge.)Kitag	3
*Densefruit Pittany Root-bark*	*Dictamnus dasycarpus* Turcz	3
*Fructus Cnidii*	*Cnidium monnieri* (L)cuss	2
*Rhizoma Smilacis Glabrae*	*Smilax glabra* Roxb	3
*Radix Rehmanniae Preparata*	*Rehmannia glutinosa* (Gaertn.) Libosch EX Fisch.et Mey	3
*Radix Angelicae Sinensis*	*Angelica sinensis* (Oliv.)Diels	2
*Pericarpium Punicae Granati*	*Punica granatum* L	3

### Ultra–High-Performance Liquid Chromatography Analysis

Standard compounds and FZHFZY dry powder were dissolved in methanol containing 0.1% formic acid. Chromatogram fingerprints were collected with an UHPLC Acquity system (Waters, United States), which comprised a UHPLC pump and a photodiode array detector scanning from 200 to 400 nm and recording at 330 nm. The HPLC conditions were as follows: column: ACQUITY BEH C18, 100 × 2.1 mm, 1.8-μm particle size (Waters), column temperature, 40°C; mobile phase: (A) acetonitrile; (B) 0.1% formic acid; flow rate, 250 μl/min; injection volume, 3 μl; gradient, 8% A (0 min), 30% A (15 min), 55% A (17 min) and 60% A (20 min).

### IL-17A/IL-22/IFN-γ/TNF-α–Stimulated HaCaT Cells

The HaCaT cell line was obtained from the Cell Culture Unit of Shanghai Science Academy (Shanghai, China). The cells were grown in DMEM supplemented with 10% FBS and incubated in a humidified 5% CO_2_ atmosphere at 37°C. Cells at 40–60% confluence were stimulated with IL-17A (10 ng/ml), IL-22 (10 ng/ml), IFN-γ (10 ng/ml) and TNF-α (25 ng/ml) for 24 h (Jiang et al., 2017; Jiang et al., 2019).

### MTT Assay

Cell viability was determined by MTT reduction assay. The cells were seeded into 96-well plates in DMEM + 10% foetal bovine serum at a density of 5,000 cells per well, and then treated with IL-17A (10 ng/ml), IL-22 (10 ng/ml), IFN-γ (10 ng/ml) and TNF-α (25 ng/ml). After incubation for 24 h, FZHFZY or DMEM (control) was added to the wells, which were then incubated for 24 h. Then, 10 μl of MTT solution was added to each well, and the plates were incubated for 4 h. Finally, the cells were lysed with 0.04 N HCl in isopropyl alcohol, and the absorbance of each well at 570 nm was assessed.

### Animals

Male BALB/c mice (6–8 weeks of age; 18–22 g) were obtained from the Experimental Animal Centre of Guangdong Province (Guangzhou, China). The mice were fed a standard diet, had free access to water and were housed under standard laboratory conditions. The animal protocols were approved by the Animal Experimental Ethics Committee of Guangdong Provincial Hospital of Chinese Medicine.

### Imiquimod-Induced Mouse Model of Psoriasis

A daily topical dose of 50 mg of IMQ cream was applied to a shaved area (3 × 2.5 cm) on the backs of the mice for 7 consecutive days, to create a psoriasis-like mouse model as previously described (Lu et al., 2019).

### FZHFZY Administration

Thirty BALB/c mice were randomly divided into five groups: 1) the control group and the IMQ group received a topical application of Vaseline; 2) the IMQ + DEX group received a topical application of 10 mg/kg of DEX; 3) the IMQ + medium-dose FZHFZY group received a topical application of 0.2 ml FZHFZY at a concentration of 0.125 g/ml once per day; and 4) the IMQ + high-dose FZHFZY group received a topical application of 0.2 ml FZHFZY at a concentration of 0.25 g/ml once per day on the shaved area on their backs. Drug administration began 2 days before the daily application of IMQ cream, and continued for 7 days.

### Psoriasis Area and Severity Index Analysis and Histological Examination of Skin

The severity of each lesion was graded and monitored using an improved human scoring system, the psoriasis area severity index (PASI), which includes the area of the skin lesions, erythema, scaling and thickening for 9 consecutive days. The PASI scores are 0 (none); 1 (light); 2 (moderate); 3 (severe); and 4 (extremely severe) (Pang et al., 2018). Tissue sections (7 μm) were cut from the paraffin sections and stained with haematoxylin and eosin (H&E) for pathological observation with an optical microscope.

### Real Time Polymerase Chain Reaction Analysis

Total RNA was extracted from the samples with Trizol (Invitrogen, United States), and the RNA concentration was detected with an ultraviolet spectrophotometer (Beckman, DU-530, United States). According to the conditions provided in the instructions for the PrimeScript RT Reagent Kit (Takara, China), after denaturation at 95°C for 15 s, annealing at 61°C for 15 s, the RNA was reverse-transcribed into cDNA *via* a total of 35 cycles of amplification. Based on the SYBR Premix Ex Taq II formulation (Takara, China), quantitative analysis was performed using a fluorescence quantitative polymerase chain reaction (PCR) instrument (Thermo Life, ViiA7, United States). The primer sequences used in this study are listed in Table 2. The relative mRNA quantities were determined by the 2^ΔΔC^T method, with data normalised to the GAPDH housekeeping gene.

**TABLE 2:** Sequence of primers.

Name	Forward	Reverse
loricrin	5′-AAG​TAA​GGT​CAC​CGG​GTT​GC-3′	5′-GGG​AAG​GGG​CGC​TTA​AAA​TG-3′
filaggrin	5′-CTA​GAG​GGC​ATG​AGT​GTA​GTC​A-3′	5′-CAA​GAC​TGG​ACA​GTT​GGC​TGG-3′
involucrin	5′-ATG​TCC​CAT​CAA​CAC​ACA​CTG-3′	5′-TGG​AGT​TGG​TTG​CTT​TGC​TTG-3′
keratin 5	5′-GTT​CCC​GGA​AGG​GAA​CGA​AT-3′	5′-AGG​GAT​GGG​GTT​CTG​CTT​TG-3′
keratin 14	5′-GCA​GTA​TCC​GAT​CTC​TTC​ATG​C-3′	5′-GGC​CCT​CGA​ATC​CTC​TGA​CT-3′
keratin 15	5′-ACC​CCA​CCA​GCA​ATG​TAG​TCT-3′	5′-CCT​GAG​AGC​GAA​TGC​CAG​A-3′
GAPDH	5′-AAG​AGG​GAT​GCT​GCC​CTT​AC-3′	5′-TAC​GGC​CAA​ATC​CGT​TCA​CA-3′

### Western Blotting

The tissues were lysed using a lysis buffer, and then centrifuged at 15,000 rpm at 4°C for 15 min to obtain the total protein samples. The total cellular protein concentration was quantified with a biscinchoninic acid kit. The proteins in each sample were resolved by 12.5% sodium dodecyl sulphate–polyacrylamide gel electrophoresis and transferred onto polyvinylidene difluoride membranes. The membranes were blocked with 3% bovine serum albumin at room temperature for 30 min. The blocked membranes were then incubated with various primary antibodies at 4°C overnight, followed by 1 h of incubation with various secondary antibodies. Finally, the proteins were detected with a Bio-Rad Imaging System (Bio-Rad Biosciences, Hercules, CA, United States).

### Statistical Analysis

The diagrams and graphs to report the cumulative data were all generated using GraphPad Prism 5.0. Statistical analyses were performed with Statistical Package for the Social Sciences software v19.0. The results are presented as means ± standard error of the mean derived from at least three independent experiments. Student’s *t*-test was used for comparisons between groups, and a one-way analysis of variance was used for multiple comparisons. A *p* value of less than 0.05 was considered to indicate statistical significance.

## Results

### Quality Control and Chemical Profiles of FZHFZY

**FIGURE 1:**
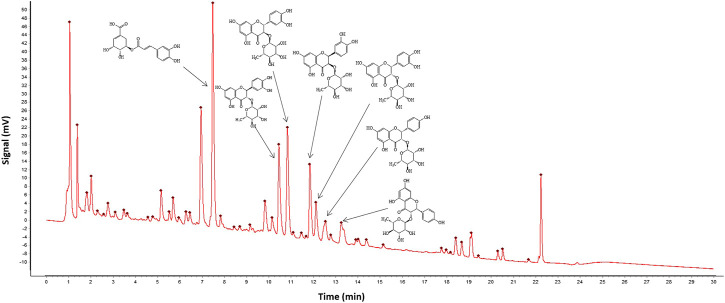
Chromatogram fingerprint was performed for quality control of seven herbs in FZHFZY.

To control the quality of FZHFZY, chromatogram fingerprinting was performed for compound analysis of FZHFZY (Figure 1). Seven chemicals, including 5-*O*-caffeoylshikimic acid, neoastilbin, taxifolin 3-*O*-rhamnoside, neoisoastilbin, and isoastilbin, were identified from the seven herbs *via* UHPLC analysis (Table 3).

**TABLE 3:** Chemical profiles of FZHFZY.

NUM.	Retention time (RT, min)	Formula	Compounds
1	7.5	C_16_H_16_O_8_	5-O-Caffeoylshikimic acid
2	10.5	C_21_H_22_O_11_	Neoastilbin
3	10.8	C_21_H_22_O_11_	Taxifolin 3-O-Rhamnoside
4	11.8	C_21_H_22_O_11_	Neoisoastilbin
5	12.1	C_21_H_22_O_11_	Isoastilbin
6	12.5	C_21_H_22_O_10_	Engeletin
7	13.4	C_21_H_22_O_10_	Isoengelitin

### FZHFZY Inhibits Proliferation and Ameliorates Epidermal Differentiation of IL-17A/IL-22/IFN-γ/TNF-α–Induced HaCaT Cells

**FIGURE 2:**
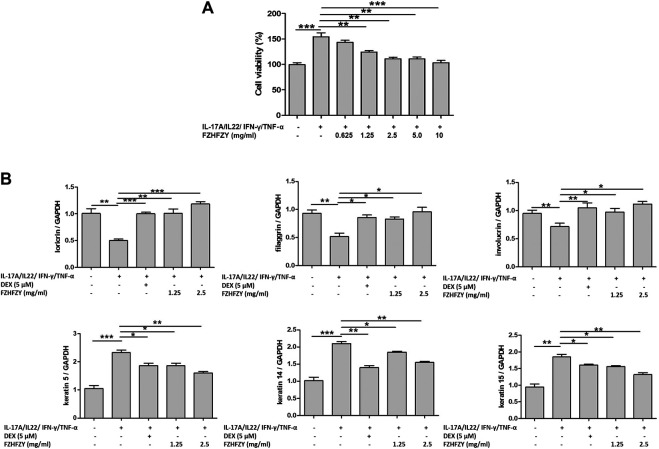
FZHFZY inhibits proliferation and improves epidermal differentiation of interleukin (IL)-17A/IL-22/interferon (IFN)-γ/tumour necrosis factor (TNF)-α–stimulated HaCaT cells. **(A)** HaCaT cells were treated with IL-17A (10 ng/ml), IL-22 (10 ng/ml), IFN-γ (10 ng/ml) and TNF-α (25 ng/ml) for 24 h, then various concentrations of FZHFZY from 0.625 to 10 mg/ml were added to the wells, followed by another 24-h incubation, and the effects of FZHFZY on cell viability was measured *via* MTT assay. **(B)** IL-17A/IL-22/IFN-γ/TNF-α–stimulated HaCaT cells were treated with 1.25 or 2.5 mg/ml FZHFZY for 24 h. Total RNA was isolated from HaCaT cells, and RT-PCR was used to investigate the mRNA levels of various molecules of epidermal differentiation.

Due to IL-17A/IL-22/IFN-γ/TNF-α–induced HaCaT cells is a preferable *in vitro* psoriatic model so far (Jiang et al., 2017; Jiang et al., 2019), we treated HaCaT cells with multiple cytokines (10 ng/ml IL-17A, 10 ng/ml IL-22, 10 ng/ml IFN-γ and 25 ng/ml TNF-α) in culture medium for 24 h to induce a psoriatic condition. We first evaluated the effects of FZHFZY on the proliferation of IL-17A/IL-22/IFN-γ/TNF-α–stimulated HaCaT cells. The viability of IL-17A/IL-22/IFN-γ/TNF-α–stimulated HaCaT cells was reduced in a dose-dependent manner after 24 h of exposure to various concentrations (0.625, 1.25, 2.5, 5 and 10 mg/ml) of FZHFZY (Figure 2A).

To evaluate the effects of FZHFZY on epidermal differentiation in IL-17A/IL-22/IFN-γ/TNF-α–stimulated HaCaT cells, we measured the mRNA expression of loricrin, filaggrin, involucrin, keratin 5, keratin 14 and keratin 15, which have recognised relationships with classical epidermal differentiation (Szczerkowska-Dobosz et al., 2020). Figure 2B shows downregulation of the mRNA levels of loricrin, filaggrin and involucrin and upregulation of the levels of keratin 5, keratin 14 and keratin 15 in IL-17A/IL-22/IFN-γ/TNF-α–induced HaCaT cells compared with the control group; FZHFZY markedly regulated the concentrations of markers related to epidermal differentiation, as indicated by RT-PCR.

### FZHFZY Improves Skin Symptoms of IMQ-Induced Psoriasis

**FIGURE 3:**
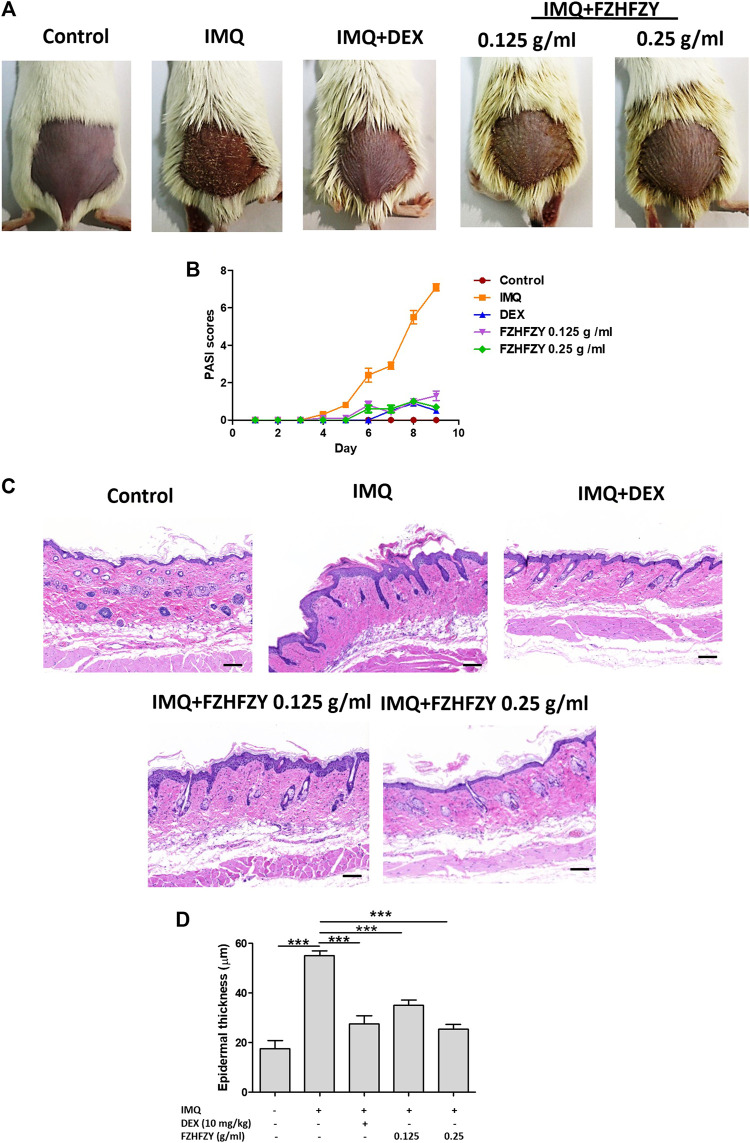
FZHFZY exerts a protective effect against imiquimod (IMQ)-induced psoriasis. **(A)** Photographs of the backs of mice on day 9 after the first FZHFZY application. All mice were randomly divided into five groups, and dexamethasone (DEX) or FZHFZY administration began 2 days before daily IMQ cream application for 7 days. **(B)** Severity of psoriasis during the course of IMQ-induced psoriasis, was evaluated by the scores on the psoriasis area and severity index (PASI). **(C)** Haematoxylin and eosin (H&E) staining of skin tissue from different groups of mice (magnification×200, scale bar = 50 μm). **(D)** Epidermal thickness of mouse dorsal skin (***p < 0.001 *vs*. IMQ-induced psoriasis group; *n* = 6).

The efficacy of external FZHFZY treatment of IMQ-induced psoriasis-like skin lesions was evaluated in a mouse model (Figure 3A), which is an ideal murine model for the study of psoriasis and its therapeutic drugs (Lu et al., 2019). The characteristic features of IMQ-induced skin inflammation (scaling, erythema and infiltration) were assigned PASI scores each day during the experiment. FZHFZY or vehicle was applied externally on the shaved areas on the backs of the BALB/c mice for 9 consecutive days, and IMQ was used from day 3 to day 9. Two days after the initiation of IMQ application, the skin on the backs of the mice began to display signs of thickening and erythema. Relative to the IMQ group, the FZHFZY-treated group showed significant reductions in specific skin scaling, erythema and infiltration from days 5–9. The PASI scores showed significant reductions with the medium dose or high dose of FZHFZY relative to the IMQ-only group (Figure 3B).

### FZHFZY Ameliorates Pathology of the Skin Tissue in IMQ Mice

To evaluate the effects of FZHFZY on mice with IMQ-induced psoriasis, a portion of the back skin of the mice in each group was fixed with 4% paraformaldehyde and then stained with H&E, as shown in Figures 3C,D. Compared with the normal control group, H&E staining showed clear histological features in the mice in the IMQ-treated group, characterised by a thickened epidermal spinous cell layer, parakeratosis and a large amount of inflammatory cell infiltration in the dermal layers. In contrast, in the DEX group and the medium-dose and high-dose FZHFZY groups, the symptoms were relieved and manifested as a reduced number of parakeratotic cells in the back skin lesions, a thinner epidermal spinous cell layer and a thinner epidermis. The results indicate that FZHFZY treatment can attenuate the psoriasis-like skin phenotype induced by IMQ.

### FZHFZY Regulates the Expression of Epidermal Differentiation-Related Molecules in the Skin of a Mice Model of Psoriasis

**FIGURE 4:**
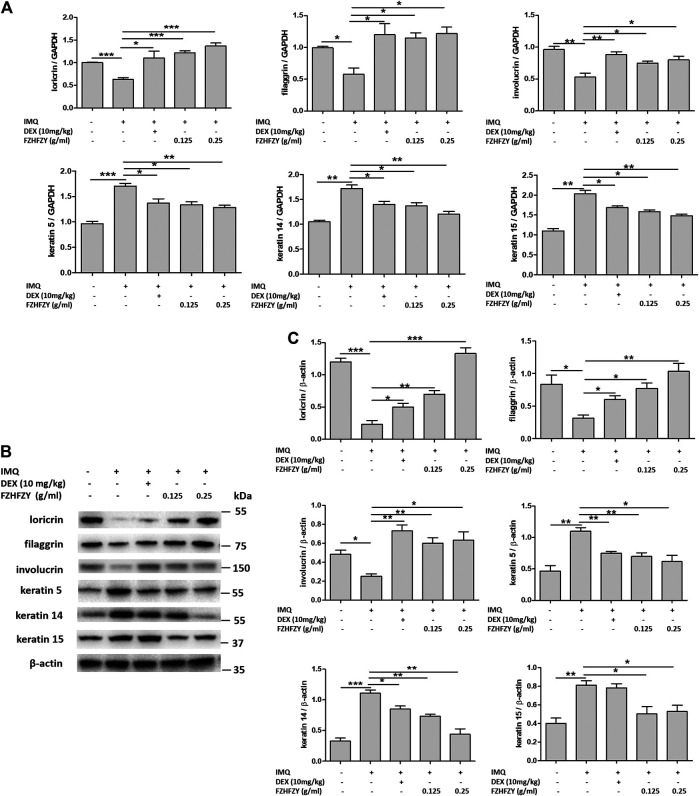
FZHFZY regulates the mRNA and protein expression of epidermal differentiation molecules in IMQ-induced psoriatic mice. **(A)** Total RNA was isolated from skin tissues, and RT-PCR was used to investigate the levels of loricrin, filaggrin, involucrin, keratin 5, keratin 14 and keratin 15. **(B)** Representative western blot of epidermal differentiation protein in the skin of mice with IMQ-induced psoriasis. **(C)** Quantification of the amounts of epidermal differentiation protein to β-actin. Data are shown as the mean ± standard error of at least three independent experiments (*p < 0.05, **p < 0.01, ***p < 0.001 *vs*. IMQ-induced psoriasis group; *n* = 6).

To assess whether FZHFZY influences epidermal differentiation in the skin of mice with IMQ-induced psoriasis, we detected the expression of loricrin, filaggrin, involucrin, keratin 5, keratin 14 and keratin 15 in the skin by RT-PCR and western blotting. The results showed that relative the control group, the expression of loricrin, filaggrin and involucrin were significantly decreased, and the expression of keratin 5, keratin 14 and keratin 15 were increased, in the IMQ group (Figure 4). However, compared with the IMQ group, there was increased expression of loricrin, filaggrin and involucrin and decreased expression of keratin 5, keratin 14 and keratin 15 in the back skin of FZHFZY groups (Figure 4). These results suggest that FZHFZY can regulate the expression of epidermal differentiation proteins in mice with IMQ-induced psoriasis.

### FZHFZY Inhibits the Akt/mTORC1/S6K1 Signalling Pathway in Skin With IMQ-Induced Psoriasis

**FIGURE 5:**
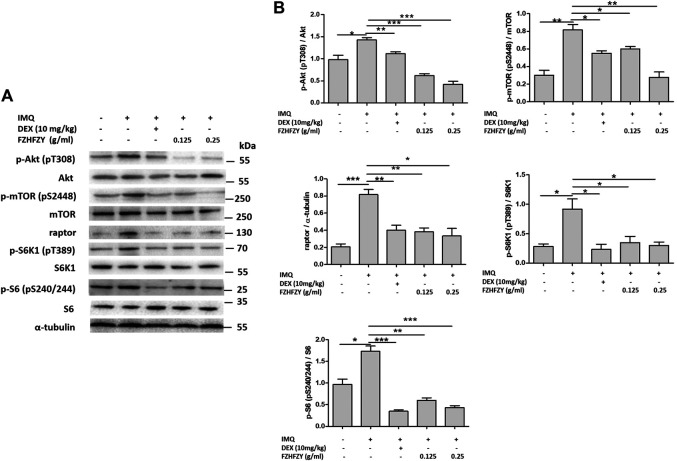
Effects of FZHFZY on the Akt/mTORC1/S6K1 signalling pathway in mice with IMQ-induced psoriasis. **(A)** Representative western blot of Akt/mTORC1/S6K1 pathway protein expression in the skin of mice with IMQ-induced psoriasis. **(B)** Quantification of the amounts of phospho-Akt, phosphor-mTOR, phosphor-S6K1, phosphor-S6 and raptor relative to those of Akt, mTOR, S6K1, S6 and α-tubulin in the skin of mice (*p < 0.05, **p < 0.01, ***p < 0.001 *vs*. IMQ-induced psoriasis group; *n* = 6).

Because the Akt/mTORC1/S6K1 pathway is closely involved in the regulation of epidermal differentiation (Akinduro et al., 2016; Buerger et al., 2017), we investigated the Akt/mTORC1/S6K1 signalling pathway in mice with IMQ-induced psoriasis. The western blot results revealed that there were dramatic increases in the proportion of p-Akt/Akt, p-mTOR/mTOR, raptor, p-S6K1/S6K1 and p-S6/S6 in mice with IMQ-induced psoriasis. Notably, the increases induced by IMQ were significantly decreased in the medium-dose and high-dose FZHFZY groups (Figure 5). Therefore, FZHFZY may suppress phosphorylation of the Akt/mTORC1/S6K1 pathway to regulate epidermal differentiation in mice with IMQ-induced psoriasis.

## Discussion

A previous study by our team demonstrated that the active components of FZHFZY, including neoastilbin, neoisoastilbin, isoastilbin and engeletin, at concentrations of 12.5–100 μg/ml, could suppress the proliferation of HaCaT cells (Chen et al., 2020). In this study, we found that FZHFZY can inhibit the proliferation of and improve the epidermal differentiation of IL-17A/IL-22/IFN-γ/TNF-α–induced HaCaT cells. We further revealed that FZHFZY significantly reduced PASI scores and decreased epidermal hyperplasia and epidermal parakeratosis compared to the IMQ-only group, thus indicating that FZHFZY relieves IMQ-induced psoriasis in mice.

Excessive proliferation and abnormal differentiation of keratinocytes were crucial for the occurrence and development of psoriasis because they resulted in thickening of the skin and an increase in scaling at the lesion site (Nestle et al., 2009). The typical histological characteristics of psoriasis are incomplete keratinisation, the excessive accumulation of stratum corneum cells, a thick spinous layer, elevated dermal papilla, a reduced or missing granular layer and infiltration with mononuclear inflammatory cells (Boehncke and Schön, 2015; Mourad et al., 2019; Sun et al., 2020). Keratinocytes express specific marker molecules at various stages during the process of epidermal differentiation (Sandilands et al., 2009). Keratin 5, keratin 14 and keratin 15 are expressed in basal keratinocytes (Bose et al., 2012; Srivastava et al., 2018); glutaminyltransferase 1 and glutaminyltransferase 5 are the marker molecules of the spinous layer (Bender et al., 2019); keratin 1, keratin 2, keratin 10, glutaminyl transferase 3 and profilaggrin are localised in the granular layer (Gschwandtner et al., 2013); and loricrin, involucrin, filaggrin, trichohyalin and S100 are expressed in the cornified layer (Rinnerthaler et al., 2015). The rapid growth of keratinocytes promotes the differentiation of the granular layer and stratum corneum, while changing the expression of keratin and related genes, which results in abnormal epidermis differentiation characterised by hypokeratosis, a thick spinous layer, a reduced granular layer and prominent epidermis (Jain et al., 2016). In our study, there is downregulated expression of loricrin, filaggrin and involucrin, but elevated expression of keratin 5, keratin 14 and keratin 15 in IL-17A/IL-22/IFN-γ/TNF-α–induced HaCaT cells and in mice with IMQ-induced psoriasis. The finding suggests that the keratinocytes of the cornified layer are not fully differentiated and the basal layer is hyperproliferative in psoriatic skin, which is consistent with the results of previous studies (Gschwandtner et al., 2013; Srivastava et al., 2018). FZHFZY plays a role in regulating epidermal differentiation, and its mechanism of action may be related to the upregulation of the expression of loricrin, filaggrin and involucrin and the downregulation of the expression of keratin 5, keratin 14 and keratin 15.

mTORC1 regulates the processes of cellular anabolism and catabolism, including autophagy, and it activates the downstream effector protein S6K1, which in turn promotes the phosphorylation of ribosomal protein S6 and perturbs keratinocyte differentiation (Jung et al., 2010; Akinduro et al., 2016). The decreased expression of phosphorylated S6K1 can promote autophagosome synthesis in normal conditions (Hac et al., 2015). The activation of mTORC1 and its downstream signalling molecules S6K1 and ribosomal protein S6 was discovered in the skin lesions of patients with psoriasis (Buerger et al., 2013; Ruf et al., 2014). In addition, the involvement of the mTORC1/S6K1 signalling pathway in the epidermal differentiation of infant mice was reported (Ding et al., 2016). Our study showed that 0.125 g/ml and 0.25 g/ml of FZHFZY could down-regulate the ratio of p-Akt/Akt, p-mTOR/mTOR, raptor, p-S6K1/S6K1 and pS6/S6 in the skin lesions of mice with IMQ-induced psoriasis, which suggests that FZHFZY regulates epidermal differentiation *via* inhibition of the Akt/mTORC1/S6K1 pathway. Our next study will focus on determining whether FZHFZY-induced inhibition of the phosphorylation of Akt/mTORC1/S6K1 can regulate the expression of epidermal differentiation-related protein in an *in vitro* model of psoriasis.

## Conclusion

Our results demonstrate that FZHFZY inhibits proliferation and ameliorates epidermal differentiation in HaCaT cells. Similarly, in mice with IMQ-induced psoriasis, FZHFZY ameliorates the symptoms of psoriasis, regulates epidermal differentiation and inhibits phosphorylation of the Akt/mTORC1/S6K1 pathway. This may be the mechanism by which FZHFZY treats psoriasis. Further investigation in an *in vitro* model of psoriasis is needed to determine the effect of FZHFZY on the relationship between epidermal differentiation and the Akt/mTORC1/S6K1 pathway.

## Data Availability

The original contributions presented in the study are included in the article/supplementary material, further inquiries can be directed to the corresponding authors.
